# Serum sample containing endogenous antibodies interfering with multiple hormone immunoassays. Laboratory strategies to detect interference

**DOI:** 10.1016/j.plabm.2015.11.001

**Published:** 2015-11-27

**Authors:** Elena García-González, Maite Aramendía, Diego Álvarez-Ballano, Pablo Trincado, Luis Rello

**Affiliations:** a Department of Clinical Biochemistry, Hospital Universitario “Miguel Servet”, Paseo Isabel La Católica 1-3, 50009 Zaragoza, Spain; b Centro Universitario de la Defensa-Academia General Militar de Zaragoza, Carretera de Huesca s/n, 50090 Zaragoza, Spain; c Department of Endocrinology and Nutrition, Hospital Universitario “Miguel Servet”, Paseo Isabel La Católica 1-3, 50009 Zaragoza, Spain

**Keywords:** ACTH, adrenocorticotropic hormone, EQAS, external quality assurance schemes, FSH, follicular stimulating hormone, EA, endogenous antibodies, fT4, free thyroxine, HCU, Hospital Clínico Universitario “Lozano Blesa”, MRI, magnetic resonance imaging, LH, luteinising hormone, PEG, polyethylene glycol, QC, quality control, TSH, thyrotropin, Endogenous antibodies, Immunoassay, Interference, Pituitary hormones, Case report

## Abstract

**Objectives:**

Endogenous antibodies (EA) may interfere with immunoassays, causing erroneous results for hormone analyses. As (in most cases) this interference arises from the assay format and most immunoassays, even from different manufacturers, are constructed in a similar way, it is possible for a single type of EA to interfere with different immunoassays. Here we describe the case of a patient whose serum sample contains EA that interfere several hormones tests. We also discuss the strategies deployed to detect interference.

**Subjects and methods:**

Over a period of four years, a 30-year-old man was subjected to a plethora of laboratory and imaging diagnostic procedures as a consequence of elevated hormone results, mainly of pituitary origin, which did not correlate with the overall clinical picture.

**Results:**

Once analytical interference was suspected, the best laboratory approaches to investigate it were sample reanalysis on an alternative platform and sample incubation with antibody blocking tubes. Construction of an in-house ‘nonsense’ sandwich assay was also a valuable strategy to confirm interference. In contrast, serial sample dilutions were of no value in our case, while polyethylene glycol (PEG) precipitation gave inconclusive results, probably due to the use of inappropriate PEG concentrations for several of the tests assayed.

**Conclusions:**

Clinicians and laboratorians must be aware of the drawbacks of immunometric assays, and alert to the possibility of EA interference when results do not fit the clinical pattern.

## 1 Introduction

**Fig. 1:**
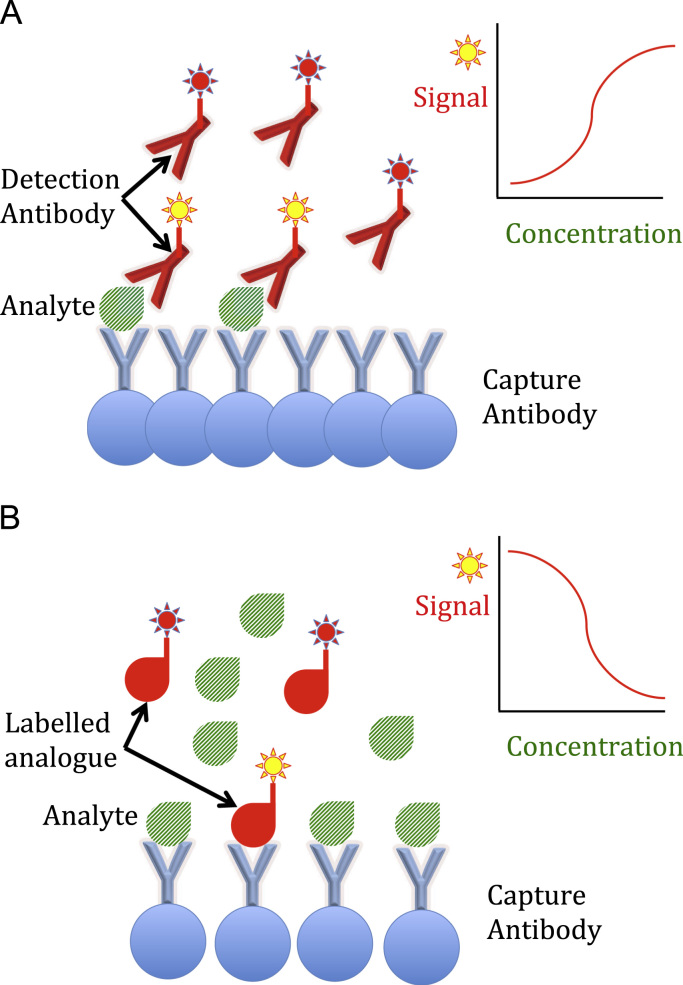
Examples of immunoassay formats. (A) Sandwich immunoassay: the reaction kit includes both capture and labelled detection antibodies that bind different epitopes of the analyte. The higher the amount of analyte, the greater the signal developed. (B) Competitive immunoassay: the reaction kit includes a capture antibody and a labelled analogue of the analyte that competes for the capture antibody. The higher the amount of analyte, the lesser the signal developed.

Two basic immunoassay formats are currently available for hormone (analyte) measurement. Total hormone quantification for large molecules is generally based on immunometric sandwich assays [1], while competitive assays are often used for analysis of free hormones and/or small molecules [2]. Examples of both types of assay format are depicted in Fig. 1, although there are many variations from the general scheme shown.

Endogenous antibodies (EA) (either heterophile antibodies, human antianimal antibodies or autoantibodies [3]) may cause interferences in these immunoassays, which often translate into erroneous tests results. Heterophile antibodies are considered to be naturally occurring because they are produced without exposure to specific immunogens. Human anti-animal antibodies are species-specific and produced following acute or chronic chronic exposure to the animal protein (immunoglobulin). Finally, autoantibodies bind specific analytes, such as anti-thyroglobulin and anti-insulin antibodies.

**Fig. 2:**
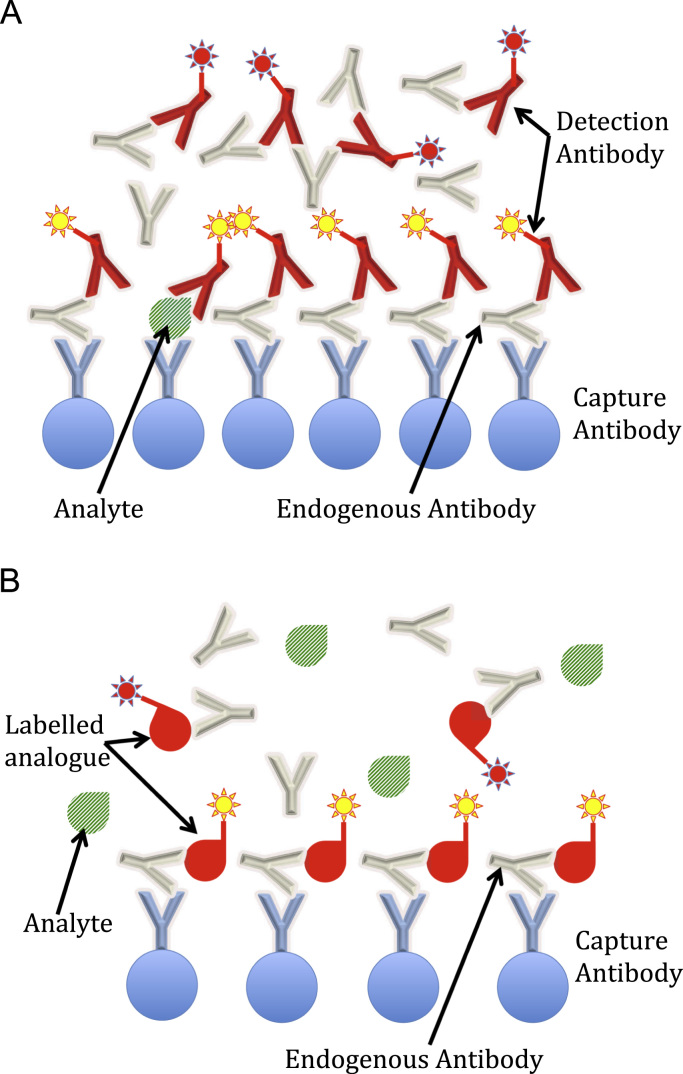
Examples of endogenous antibodies (EA) interference in immunoassays. (A) Sandwich immunoassay. EA can bind both the capture and the detection antibodies. In the case depicted, interference causes a falsely elevated result. (B) Competitive immunoassay. The example shows EA binding both the capture antibody and only the labelled analogue (but not the analyte). In this case, interference will produce a falsely low result.

The mechanism by which EA cause interference is different depending on the type of antibody and the immunoassay format. Heterophile and anti-animal antibodies, for instance, usually work by cross-linking capture antibodies with detection antibodies in the absence of antigen (that is, the analyte to be measured), thus resulting in a false positive result (although also false negative results are possible, depending on the assay construction and the site of interference). Therefore, this kind of interference is more common with sandwich assay protocols (see Fig. 2A). However, although more rarely reported, even competitive assays may suffer from the presence of EA, as depicted in Fig. 2B. Interestingly, it appears that current competitive immunoassays may be more susceptible to EA interference than older competitive radioimmunoassays, due to the combination of components used in today's reagents [2].

It has been reported that up to 40% of people may have antibodies with affinity to animal antibodies, although it is thought that most of them will not create problems in immunoassays [1]. However, this kind of interference, along with others derived from preanalytical factors (e.g., physiological factors, medications, adherence to sample collection and sample preservation protocols, etc.), must be always taken into account to ensure correct interpretation of a test result. Unfortunately, modern practice in clinical laboratories demands dealing with thousands of samples every day, so it is difficult for laboratory specialists to detect some of these aberrant results, especially when most request forms received do not provide relevant clinical information.

Laboratories try to ensure the quality of their results by minimising the effect of those preanalytical variables that can be directly controlled from the laboratory, but also by participating in external quality assurance schemes (EQAS), which are mainly focused on analytical aspects (the “measuring process”). However, a perfect EQAS performance will usually not identify assay methods that are susceptible to patient-specific interferences, such as those derived from the presence of EA [4]. In most cases, laboratorians examine test results by applying certain rules to detect inconsistencies. For example, unusual changes in the pattern of results with respect to previous data (“delta checks”) for an individual whose clinical situation has not changed, or discrepancies in the results obtained for hormones directly related to each other (e.g. low free thyroxine (fT4) values with normal thyrotropin (TSH) values). However, these checks will fail to detect spurious results that, a priori, are perfectly possible, such as high TSH with normal free thyroxine (fT4) values (suggesting subclinical hypothyroidism), individual elevations in tumour markers (suggesting neoplasia) or an initial high troponin value in an emergency ward patient (suggesting acute myocardial infarction). All of these [5], [6], [7], [8], along with many more situations, have been described to occur with almost any analytical platform [9], with dramatic consequences for the patients in some cases [1].

In view of all the evidence of interferences in immunoassays, it is somewhat surprising that some medical procedures (either invasive procedures or a drug prescriptions) are still performed based solely on an isolated laboratory immunoassay result, which has not been confirmed by other diagnostic methods and which does not fit the overall clinical picture for the patient under consideration. This is probably because many clinicians are unaware of the pitfalls that can befall immunoassays and have blind (and misplaced) faith in the integrity and authenticity of laboratory data. This is aggravated by the present tendency (arising from high-volume automated testing, unrestricted electronic requesting and/or defensive medicine protocols often instituted by healthcare practitioners) to request multiple tests with the aim of ruling out pathologies which are not clinically suspected and which, therefore, are not necessary for patient evaluation [10].

Although reporting and interpreting erroneous results is the responsibility of the laboratory [11], it is often the clinician who identifies these results [4]. Good communication and collaboration between the clinician and the laboratory specialist to identify and evaluate discordant results is thus essential and, in such circumstances, the laboratory needs to have a range of approaches to assess the presence of interfering substances [1], [2], [11], [12], [13].

In this paper we present the case of an asymptomatic ambulatory patient with EA in whom spurious hormone results first led to the misdiagnosis of subclinical hypothyroidism and later to that of possible pituitary adenoma. He was eventually referred to one of us (D. A-B), who was aware of the potential drawbacks of analytical methods and suspected that an interference was responsible for the discrepancy between some of the hormone results and the rest of the diagnostic procedures carried out. The relevance of this case lies in the fact that EA interference affected multiple hormone tests, most of them of pituitary origin, a situation that has rarely been reported with the exception of a similar case recently described by Gulbahar et al. [14]. Both studies seem to indicate that the number of samples susceptible to multiple interferences in immunoassays may be greater than suspected.

## 2 Case presentation

In October 2011, a 30-year-old man consulted his General Practitioner complaining of prolonged diarrhoea. His past medical history was unremarkable. He was subjected to faecal, urine and blood analysis and all results were negative with the exception of a relatively high TSH (16.97 μU/mL, reference interval 0.34–5.60) while the fT4 was within the reference range. Anti-peroxidase and anti-thyroglobulin antibodies were negative. One month later, these thyroid function test results were further confirmed by two repeat analyses. The patient did not have any personal or family history of known thyroid dysfunction. There was no goitre on examination. Neck ultrasonography showed an average-sized thyroid gland and an echo structure with no obvious abnormalities, no nodules and normal vascular flow.

Despite the fact that the patient showed no clinical signs or symptoms of hypothyroidism and the unlikely possibility (below 1%) of a young male presenting with such a medical condition [15], he was diagnosed with subclinical hypothyroidism and prescribed 50 micrograms of Levothyroxine per day. Surprisingly, his serum TSH level did not decrease with the treatment but, in fact, it increased steadily (Table 1), despite the fact that his Levothyroxine dose was increased progressively to 200 micrograms per day. Patient non-compliance was initially suspected, but this was discounted. To rule out intestinal malabsorption, markers for celiac disease were requested and they were all negative. He was not taking any other drugs that might have interfered with the Levothyroxine absorption. Importantly, since having been prescribed 200 micrograms of Levothyroxine per day, the patient complained of headaches and insomnia but no weight loss or palpitations.

**Table 1:** Patient cumulative laboratory results. Results exhibiting interference are shaded.

**Assay**^a^**/units (reference range)**	**20/10/2011**	**23/11/2011**	**16/2/2012**	**22/5/2012**	**18/6/2012**	**25/1/2013**	**21/11/2013**	**19/2/2015**	**19 /2/2015 (HCU lab)**^b^
**TSH** – µU/mL (0.34–5.60)	16.97**	17.73**	21.33**	26.91**	35.97**	14.90**	8.36*	10.23*	**0.05 (0.55–4.78)
**fT4** – ng/dL (0.58–1.64)	0.93	0.93	0.83	0.96	1.19	1.35	1.18	0.84	1.19 (0.89–1.76)
**fT3** – pg/mL (2.39–4.01)						4.44*		3.22	3.19 (2.3–4.2)
**Ab. TPO** – U/mL (0–4)		0.6	0.7			0.3		0.7	11.98 (0–100)
**Ab. Tg** – U/mL (0–9)						0.6		1.5	4.47 (0–138)
**LH** – mU/mL (1.26–10.05)						290.62**		128.54**	4.88 (0.8–7.6)
**FSH** – mU/mL (1.27–19.26)						87.52**		60.35**	2.16 (1.5–14)
**Prolactin** – ng/mL (2.64–13.3)						75.27**	54.12**	45.37**	8.96 (2.5–17)
**Oestradiol** – pg/mL (11–44)						31		19	43 (<15–56)
**ACTH** – pg/mL (5–46)					583**	396**	209**	165**	143**
**Cortisol** – µg/dL (5.0–25.0)					15.98	12.75	11.65	5.09	*4.06 (5–25)
**GH** – ng/mL (0.15–3.00)								0.42	0.44
**IGF-1** – ng/mL (115–307)						322*		403*	244
**Testosterone** – ng/mL (1.75–7.81)						4.82	4.22	3.81	**0.41 (1.6–7.26)
**Free testosterone** – pg/mL (2.60–9.79)								4.56	8.48 (4.25–30.37)
**SHBG** – nmol/L (13.5–71.4)								41.7	45.3 (10–57)
**Glycoprotein hormone alpha-subunit** – mU/mL								<0.3 (0–1.6)	

X*: above the upper reference limit. X**: well above the upper reference limit. *X: below the low reference limit. **X: well below the low reference limit.

a TSH: thyrotropin; fT4: free thyroxine; fT3; free triiodothyronine; Ab. TPO: anti-peroxidase antibodies; Ab. Tg: anti-thyroglobulin antibodies; LH: luteinising hormone; FSH: follicular stimulating hormone; ACTH: adrenocorticotropic hormone; IGF-1: insulin-like growth factor 1; GH: somatotropin; SHBG: sex hormone-binding globulin.

b The results obtained in HCU laboratory are accompanied by their reference values when the analytical methods employed were different from those used at our laboratory.

In June 2012 he presented again with a persistently elevated result for TSH. To rule out Addison's disease (in which a elevated TSH values are found in some untreated patients [16]) a request for adrenocorticotropic hormone (ACTH) and cortisol determination was received in the laboratory, and elevated results were obtained for the ACTH. Cushing's disease (due to an ACTH secreting pituitary tumour) was excluded by a low dose dexamethasone suppression test and Addison's disease (adrenocortical insufficiency) was excluded by normal results for a short Synacthen test.

In January 2013, a repeat complete hormonal study was requested. Results above the upper reference limit were obtained not only for TSH and ACTH, but also for luteinising hormone (LH), follicular stimulating hormone (FSH) and prolactin (see Table 1). As a result of the abnormal endocrine profile, several diagnostic imaging procedures were ordered to exclude the possibility of a pituitary tumour. Unfortunately, the patient missed the next scheduled clinic visit and was next seen in February 2015. Repeat endocrine profile analysis confirmed the previous results. This profile also included measurement of glycoprotein hormone alpha-subunit, which was normal. A pituitary magnetic resonance imaging (MRI) scan was also performed and this was normal.

At that time, a new endocrinologist (author D. A-B) reviewed the patient's clinical history. Given the persistence of discordant values between pituitary and peripheral hormones, he suspected the presence of EA in the patient sera. When questioned about possible contacts with animals, the patient confirmed that he had lived and worked on a farm during his childhood and adolescence, where he had direct contact with various animal species, including goats, sheep, cows, rabbits and pigs.

## 3 Materials and methods

Blood samples from the patient were drawn following a standard phlebotomy technique and were analyzed immediately in our laboratory using the techniques and manufacturers detailed in Table 2. Unless otherwise indicated, all assays were performed on serum samples, except for ACTH, for which EDTA plasma is the specimen of choice for our method.

**Table 2:** Assays carried out with the patient's samples. We include the species of the reagent antibodies (Abs) (capture and detection Ab in sandwich-type immunoassays or only capture Ab in competitive immunoassays), the assay format and the platform employed for analysis (instrument and manufacturer). The assays affected by the presence of endogenous antibodies are shaded. Assay abbreviations are as Table 1.

**Assay**	**Our laboratory**			**HCU laboratory**		
	**Reagent Abs**^a^	**Type**	**Platform**^b^	**Reagent Abs**	**Type**	**Platform**^b^
**TSH**	goat–mouse/goat	Sandwich	Dxi-BC	mouse/sheep	Sandwich	Adv-S
**fT4**	mouse	Competitive	Dxi-BC	mouse	Competitive	Adv-S
**fT3**	?	Competitive	Dxi-BC	rabbit	Competitive	Adv-S
**Ab. anti TPO**	–	Sandwich	Dxi-BC	goat	Sandwich	Inova
**Ab. anti Tg**	–	Sandwich	Dxi-BC	goat	Sandwich	Inova
**LH**	goat–mouse/goat	Sandwich	Dxi-BC	mouse/goat	Sandwich	Imm-S
**FSH**	goat–mouse/goat	Sandwich	Dxi-BC	mouse/mouse	Sandwich	Imm-S
**Prolactin**	goat–mouse/goat	Sandwich	Dxi-BC	mouse/goat	Sandwich	Imm-S
**Oestradiol**	mouse	Competitive	Arc-A	rabbit	Competitive	Imm-S
**ACTH**	mouse/rabbit	Sandwich	Imm-S	mouse/rabbit	Sandwich	Imm-S
**Cortisol**	mouse	Competitive	Arc-A	rabbit	Competitive	Imm-S
**GH**	mouse/rabbit	Sandwich	Imm-S	mouse/rabbit	Sandwich	Imm-S
**IGF-1**	mouse/rabbit	Sandwich	Imm-S	mouse/rabbit	Sandwich	Imm-S
**Testosterone**	goat–mouse	Competitive	Dxi-BC	rabbit	Competitive	Imm-S
**Free testosterone**	mouse	Competitive	IBL	rabbit	Competitive	DBC
**SHBG**	mouse/mouse	Sandwich	Arc-A	mouse/rabbit	Sandwich	Imm-S

a ?:not specified. –: Antibody not included in the reagent kit.

b Dxi-BC, Dxi-Beckman Coulter (Barcelona, Spain); Imm-S, Immulite-Siemens Healthcare Diagnostics (Erlangen, Germany); Adv-S, Advia Centaur-Siemens Healthcare Diagnostics; Arc-A, Architect-Abbott Diagnostics (Wiesbaden, Germany); Inova, Inova diagnostics (San Diego, CA, USA); IBL, IBL International (Hamburg, Germany); DBC, Diagnostics Biochem (Dorchester, ON, Canada).

The remainder of the experiments detailed below were only carried out once interference was suspected.

A series of serial dilution assays were made by dilution of the sample (2, 3 and 5-fold) using the manufacturers’ diluents.

Sample treatment with polyethylene glycol (PEG) was made by mixing equals parts of serum or plasma samples with a 25% (w/v) buffered phosphate solution of PEG 6000 (Merck, Darmstadt, Germany; reference 817007), prepared as described by Sturgeon and Viljoen [13].

For comparison purposes, aliquots of the patient's samples were sent to a nearby hospital (Hospital Clínico Universitario “Lozano Blesa”, Zaragoza, Spain [HCU]) for hormone quantification by means of alternative analytical methods (whenever possible), as detailed also in Table 2.

Analysis to confirm the presence of EA was made using a mix of interference-eliminating proteins provided by Beckman Coulter (Barcelona, Spain), composed of PolyMAK-33 (Roche Diagnostics, Mannheim, Germany), HBR-1 (Scantibodies Laboratory, Santee, CA, USA) and goat, mouse, rabbit and bovine immunoglobulins.

An in-house ‘nonsense’ sandwich assay was constructed by replacing reagent 1 (R1) from a TSH Beckman Coulter reagent (reference 33820) with the R1 from a prolactin Beckman Coulter reagent (reference 33530). In this way, capture and signal antibodies have different specificity and the only way that the assay can produce a signal above blank levels is in the presence of an interfering substance that cross-links both reagent antibodies (prolactin R1 and TSH R2).

We have obtained informed written consent from the patient for publication of this work.

## 4 Results and discussion

When it is suspected that a sample contains endogenous interfering antibodies, repeat analysis using an alternative method/instrument (and therefore, with different reagent antibodies and/or assay design) will solve the interference in most cases. However, several further experiments are available to most laboratories to try to demonstrate the presence of EA [1], [2], [11], [12], [13]. One of the simplest solutions is sample dilution with the manufacturer's diluent solution, since a nonlinear relationship is often obtained for serial dilutions in the presence of EA. Also, sample treatment with PEG will precipitate serum immunoglobulins and, therefore, EA. Nowadays, however, the best approach to demonstrate EA interference may be the use of commercially available antibody blocking tubes containing serum proteins and immunoglobulins from several animal species. EA in serum samples will preferentially bind to the serum proteins and immunoglobulins from these blocking tubes, thus allowing the reagent antibodies to bind the sample analyte. Finally, in-house nonsense sandwich assays (constructed with two reagent antibodies with reactivity against different analytes or, alternatively, using the same reagent antibody both as capture and detection antibody [17]) would only provide a positive test result in the presence of endogenous substances that cross-link the reagent antibodies. All of these experiments were performed with our patient's serum with different outcomes as detailed below.

When we subjected the sample to serial dilutions, a linear relationship was obtained for all hormones analyzed, which seemed to indicate that the original results were correct. However, despite the simplicity of this confirmatory test, it has been reported that approximately 40% of samples containing interfering antibodies fail to show a nonlinear relationship in the serial dilution test [18], as occurred in our case.

In the same way, precipitation of samples with PEG provided inconclusive results. We used a 25% (w/v) phosphate buffered solution of PEG 6000, since this is the concentration we routinely use to evaluate the presence of macroprolactin [13], [19]. We performed all hormone tests with the supernatant obtained with these PEG-treated sera (or EDTA plasma in the case of ACTH). As shown in Table 3, FSH and prolactin results after PEG precipitation seemed to suggest interference but results were difficult to interpret for the rest of hormones analyzed since matrix effects were clearly present. Thus, it was not possible to obtain any result for LH (and only a few results for TSH) on PEG-treated samples. We speculate that the PEG which does not precipitate after centrifugation and, therefore, remains in the serum supernatant, provides a high-density sample incompatible with some of the chemiluminescent assays, although why this effect occurred with some assays and not with others employing the same methodology, is not clear.

**Table 3:** Effect of PEG precipitation on the results obtained for samples of several patients (Numbers in column 1 are laboratory ID numbers for other patients studied. The case described in the text is at the top of the Table).

	**Sample**	**TSH** (µU/mL)	**fT4** (ng/dL)	**LH** (mU/mL)	**FSH** (mU/mL)	**Prolactin** (ng/mL)	**Testosterone** (ng/mL)	**ACTH** (ng/mL)
Case Patient	untreated	7.49	0.95	103.26	45.28	36.4	3.83	
serum	+PEG	x^a^	2.48	x	1.76	4.16	3.14	
Case Patient	untreated	5.96	0.89	91.87	54.5	33.86	8.53	135
EDTA^b^	+PEG	1.50	x	x	2.44	3.96	6.02	<10
1134669	untreated	1.87	0.86	34.15	15.70	8.44	0.00	
serum	+PEG	x	2.14	x	13.50	9.06	x	
1134669	untreated	1.43	0.90	33.73	15.95	7.92	0.75	20.3
EDTA	+PEG	4.90	2.24	x	15.38	9.76	0.66	<10
1236183	untreated	1.41	0.70	8.50	6.28	24.74	0.30	
serum	+PEG	2.82	2.00	x	3.96	24.86	0.00	
1236183	untreated	0.81	0.70	9.03	6.72	23.88	0.94	13.9
EDTA	+PEG	x	1.80	x	6.18	27.32	0.98	<10
6497661	untreated	4.02	0.94	7.85	4.90	15.23	0.46	
serum	+PEG	x	2.52	x	3.78	14.36	0.00	
6497661	untreated	2.60	0.98	8.17	6.09	14.81	1.34	14.7
EDTA	+PEG	5.80	2.61	x	5.58	15.06	1.62	x
6549919	untreated	0.36	1.15	62.62	104.42	10.86	0.65	
serum	+PEG	x	3.08	x	83.44	10.60	0.24	
6363715	untreated						8.72	
serum	+PEG						7.04	
6551911	untreated	1.13	2.37	6.03	15.28	9.49	3.64	
serum	+PEG	2.64	5.06	x	16.20	11.60	3.20	

TSH, fT4, LH, FSH, prolactin and testosterone assays were performed on the Dxi Beckman Coulter analyzer and ACTH on the Immulite Siemens analyzer.

a x denotes assays showing interference by the presence of PEG in the sample supernatant.

b The recommended samples for all assays performed with the Beckman Coulter Access® instrumentation are serum or heparinized plasma.

The results that we obtained for LH after PEG precipitation contradict those reported by Ellis and Livesey [20] who assayed LH, FSH, and prolactin using the same instrumentation as we did. They did not report any methodological difficulties, although the median LH recovery after PEG precipitation was 53%. In contrast, they reported recoveries around 100% for FSH and prolactin, values close to our data (see Table 3). At this point, we investigated if some methodological differences in sample treatment could explain these discrepant results for LH. Ellis and Livesey used a 25% (w/v) phosphate buffered PEG solution as well, which, however, contained also 0.5 g/L of Triton X-100. Furthermore, they employed EDTA plasma samples, whereas we used sera, as only serum or heparinized plasma are the samples recommended by the manufacturer. Despite this, we conducted additional experiments using EDTA plasma to check if this specimen could suppress the PEG matrix effects observed in some of the assays. In PEG-untreated samples, serum and EDTA results correlated reasonably well for fT4, LH, FSH and prolactin, whereas important differences were obtained for TSH and testosterone. In any case, PEG precipitation provided similar results with serum and EDTA samples only for FSH and prolactin. Most importantly, LH assay continued to be show matrix interference with PEG-treated EDTA samples in all cases.

This dissimilar behaviour observed for different immunoassays after PEG precipitation has already been highlighted in several publications [11], [13], [20], [21]. The conclusion after reviewing these papers is that employing a single PEG concentration is probably not a robust strategy when several tests need to be evaluated. The PEG concentration used for assessing macroprolactin (and generally used in most clinical laboratories) must be carefully re-evaluated before application to other analyte/instrumentation combinations. However, since in the case we studied many hormones were suspected of EA interference, we considered that trying to fine-tune the individual PEG concentration for each analyte was not a very profitable strategy, as the other experiments that were carried out (see below) clearly demonstrated interference.

At the same time as the serial dilution and PEG precipitation tests were being conducted, sample aliquots were sent to the HCU laboratory, where analysis with alternative methods (whenever possible) was repeated for most hormones (Table 2). As can be seen in Table 1, completely normal values were obtained for most hormones. For ACTH a comparably high value was obtained, which could be expected since both laboratories use the same analytical platform for this parameter.

Patient sample aliquots were also sent to Beckman Coulter facilities to be treated with a mix of blocking substances: PolyMAK-33, HBR-1 and goat, mouse, rabbit and bovine immunoglobulins. Using immunoglobulins from multiple animal species increases the probability of removing the interference from EA, especially considering that in our case several assays with reagent antibodies from different species (mouse, goat and rabbit) seemed to be affected. In fact, the use of blocking substances against only one species can be ineffective [22]. The results of this experiment are summarized in Table 4. Recovery for a high quality control (QC) sample was around 100 (±20)% while recoveries for the patient's sample were clearly diminished by the addition of blockers. Therefore, it was confirmed that this sample contained an interfering substance causing falsely elevated results with the TSH, LH, FSH and prolactin assays on the Beckman Coulter Access system. All these assays have the same construction format, with reagent antibodies derived from the same species (see Table 2).

**Table 4:** Effect of the addition of blocking agents on the results of the Beckman Coulter Access® Beckman Coulter hormone assays suspected of interference by endogenous antibodies.

**Assay**	**Blocker**	**Patient sample**		**QC sample**	
		**Result**	**Recovery (%)**	**Result**	**Recovery (%)**
**TSH** (mUI/mL)	Untreated	12.86	–	26.19	–
	+Pool 1	0.61	4.7	28.63	109.3
	+Pool 2	1.07	8.3	30.13	115.0
**LH** (mUI/mL)	Untreated	121.80	–	66.54	–
	+Pool 1	23.42	19.2	51.43	77.3
	+Pool 2	21.49	17.6	61.67	92.7
**FSH** (µUI/mL)	Untreated	47.46	–	42.57	–
	+Pool 1	7.96	16.8	34.44	80.9
	+Pool 2	6.61	13.9	39.80	93.5
**Prolactin** (ng/mL)	Untreated	39.87	–	43.17	–
	+Pool 1	8.24	20.7	40.13	92.9
	+Pool 2	11.55	29.0	41.68	96.6

Pool 1 and pool 2 were mixtures of different blockers: PolyMAK-33, HBR-1 and goat, mouse, rabbit and bovine immunoglobulins (see text). The manufacturer did not reveal the exact composition of each of the pools.

It is worth mentioning at this point that the addition of the mix of blockers did not completely eliminate the interference for TSH and prolactin. Measured TSH concentration decreased to values within the reference range, which would still have led to misdiagnosis since in fact, and as confirmed in the HCU laboratory, the true concentration was very low (0.05 μU/mL). This was expected as this patient had been treated for 4 years with high doses of Levothyroxine, which had resulted in an iatrogenic hyperthyroid state. It has been reported that treatment with blocking substances can be ineffectual in demonstrating interference in 20–30% of samples known to contain endogenous interfering antibodies [11]. Moreover, and even when interference is proved, this treatment might be insufficient to completely remove the interference, as in our case.

To further confirm the presence of an interfering substance, we made use of an in-house ‘nonsense’ sandwich assay (see Section 3). In this assay, capture and signal antibodies had different analyte specificity and the only way that the assay could provide signal readings above blank levels was in the presence of an interfering substance that cross-linked both reagent antibodies [17]. The results of this experiment are shown in Table 5. As can be seen, the TSH assay in the ‘nonsense’ format provided a similarly high result only with the patient sample. In contrast, the results obtained with other patients’ or QC samples were at the blank level (or markedly reduced, as for the high QC sample). It is fair to stress at this point that extrapolating the use of this simple strategy in similar situations is probably not possible for all cases, depending on the particular interference, assay format and/or analytical platform. However, most clinical laboratories do not have the means to constructing the type of ‘nonsense’ assays described in literature as, for example, by Bjerner et al. [[17] and references therein] or by Bolstad et al. [22]

**Table 5:** Results obtained with the TSH Beckman Coulter Access® assay, both in the normal format (with the reagent kit provided by the manufacturer) and with the ‘nonsense’ in-house format (see text). Patient case is the patient described in the text. Other patients studied are identified by laboratory ID number.

**Sample**	**Normal TSH assay (µU/mL)**	**‘Nonsense’ TSH assay (µU/mL)**
QC low level	0.65	<0.003
QC high level	25.6	0.12
Patient case	7.49	8.38
Patient 1134320	3.60	0.01
Patient 6461018	1.12	<0.003

After discussion of these results with the endocrinologists, it was decided to stop treatment and not to perform any further diagnostic procedures. Four months later, a new blood analysis in both laboratories gave similar results to the previous ones (results not shown), although the TSH value in the HCU laboratory (without interference) had already normalized, due to discontinuation of thyroxine treatment.

At that time, antibody blocking tubes (HBT) were available in our laboratory, which allowed us to conduct in-house experiments with blocking substances to assess EA interference in all assays performed. Thus, interference in the TSH, FSH, LH and prolactin Beckman Coulter Access assays were again confirmed, and additionally, it was also demonstrated in the ACTH, insulin-like growth factor 1 and total testosterone Immulite (Siemens) assays. Interestingly, the total testosterone Immulite assay was the only competitive assay that exhibited interference, giving a falsely low result. The remainder of the assays that showed interference were of the sandwich type and gave falsely elevated results.

This information was recorded in the patient's clinical file and an alert was included to recommend clinicians to interpret any laboratory result with great care, in the light of the potential risk of erroneous results caused by EA interference

This case clearly shows the problems (both clinical and financial) caused by undetected interference in immunoassays. The patient was unnecessarily treated with a high dose of Levothyroxine for four years and, during this period, he was subjected to 21 blood analysis, 405 laboratory tests (including two dynamic function tests) and four diagnostic imaging procedures. Additionally, he had 19 clinical consultations with two different general practitioners and three different endocrinologists, of which only the last one (author D. A-B) suspected analytical interference. Clearly, therefore, every effort should be made to increase clinician awareness of this problem, as it is the requesting clinician who is best placed to detect inconsistencies between the laboratory results and the patient's clinical state [4]. For instance, if the first general practitioner who saw the patient had been familiar with the possibility of misleading immunoassay results, it is likely that a high TSH result in a young man would have aroused suspicion, as the prevalence of subclinical hypothyroidism in this population is less than 1% [15]. In fact, Ismail *et al*. [23] estimated that the probability of a raised TSH result in a young adult being a false positive is greater than 30%.

Early communication between clinicians and laboratorians is thus of paramount importance, so that any laboratory result not correlating with the patient clinical presentation can be investigated further. If analytical interference is suspected, the laboratory can then carry out extra confirmatory tests such as those described above, which are relatively simple and inexpensive [23].

The mechanism of interference by endogenous antibodies is complex and speculative, and varies from one patient to another [11]. In our case, discovery of the exact nature of the interference was not pursued. However, since interference affects several assays with different reagent species (goat, mouse and rabbit), one can speculate that the patient generated antibodies against immunoglobulins of these species, as a consequence of his living on a farm. Antibodies against antibodies can be of the anti-isotypic, anti-allotypic or anti-idiotypic type. Endogenous anti-idiotypic antibodies would be highly specific for one particular immunoglobulin and, therefore, for just one assay and one platform [14]. The presence of endogenous anti-isotypic or anti-allotypic antibodies in the patient's sera may explain why some assays show interference and some do not, despite the use of reagent antibodies from the same species. However, this can only be speculation, since the exact isotype and allotype of the reagent antibodies are not revealed by the manufacturers. In any case, discovering the exact nature of interference can be challenging even for laboratories that have the resources to investigate it.

## 5 Conclusions

This case report demonstrates several learning points: (i) any laboratory result can be erroneous and, in particular, test results that conflict with the overall clinical picture should be further investigated, (ii) clinicians must be especially aware of the drawbacks of immunometric assays and spurious results caused by endogenous antibodies interference may be more frequent than suspected, and (iii) communication between the requesting clinician and the laboratory is vital to avoid erroneous laboratory results translating into harmful consequences for patients.

Once interference is suspected, several approaches (such as the ones presented in this paper) should be available to most laboratories to detect and confirm it: i.e. serial dilutions, PEG precipitation, antibody blocking tubes, alternative analytical platforms and in-house ‘nonsense’ assays.
